# Effects of Dietary Zinc Sources on Growth Performance and Gut Health of Weaned Piglets

**DOI:** 10.3389/fmicb.2021.771617

**Published:** 2021-10-28

**Authors:** Hui Diao, Jiayou Yan, Shuwei Li, Shengyao Kuang, Xiaolan Wei, Mengjia Zhou, Jinxiu Zhang, Chongbo Huang, Peng He, Wenjie Tang

**Affiliations:** ^1^Animal Breeding and Genetics Key Laboratory of Sichuan Province, Sichuan Academy of Animal Science, Chengdu, China; ^2^Sichuan Animtech Biology Development Co., Ltd, Chengdu, China; ^3^Livestock and Poultry Biological Products Key Laboratory of Sichuan Province, Sichuan Animtech Feed Co., Ltd, Chengdu, China

**Keywords:** zinc source, growth performance, digestibility, gut health, weaned piglets

## Abstract

The present study aimed to investigate the effects of dietary zinc sources on the growth performance and gut health of weaned piglets. In total, 96 Duroc × Landrace × Yorkshire (DLY) weaned piglets with an initial average body weight of 8.81±0.42kg were divided into four groups, with six replicates per treatment and four pigs per replicate. The dietary treatment groups were as follows: (1) control group, basal diet; (2) zinc sulphate (ZnSO4) group, basal diet +100mg/kg ZnSO4; (3) glycine zinc (Gly-Zn) group, basal diet +100mg/kg Gly-Zn and (4) zinc lactate group, and basal diet +100mg/kg zinc lactate. The whole trial lasted for 28days. Decreased F/G was noted in the Gly-Zn and zinc lactate groups (*p*<0.05). The zinc lactate group had a lower diarrhea rate than the control group (*p*<0.05). Moreover, the ZnSO4, Gly-Zn, and zinc lactate groups had significantly higher apparent total tract digestibility of dry matter (DM), crude protein (CP), ether extract (EE), crude ash, and zinc than the control group (*p*<0.05). The Gly-Zn and zinc lactate groups had higher jejunal villus height and a higher villus height:crypt depth ratio than the control group (*p*<0.05). In addition, the ZnSO4, Gly-Zn and zinc lactate groups had a significantly lower mRNA expression level of jejunal ZRT/IRT-like protein 4 (ZIP4) and higher mRNA expression level of jejunal interleukin-1β (IL-1β) than the control group (*p*<0.05). The mRNA expression level of jejunal zinc transporter 2 (ZNT2) was higher and that of jejunal Bcl-2-associated X protein (Bax) was lower in the Gly-Zn and zinc lactate groups than in the control group (*p*<0.05). Moreover, the zinc lactate group had a higher count of *Lactobacillus* spp. in the cecal digesta and higher mRNA expression levels of jejunal occludin and mucin 2 (MUC2) than the control group (*p*<0.05). In conclusion, dietary supplementation with 100mg/kg ZnSO4, Gly-Zn, or zinc lactate could improve the growth performance and gut barrier function of weaned piglets. Dietary supplementation with organic zinc, particularly zinc lactate, had the best effect.

## Introduction

In the weaning stage, piglets have weak cerebral cortex development and high metabolism and face high nutritional, environmental, and psychological stress, resulting in changes in intestinal digestion and absorption, immunity, and behavior, which manifest as diarrhea, growth retardation, and even death (Campbell et al., 2013). In recent years, weaning stress of piglets has been found to damage the intestinal mucosal barrier function of piglets and their innate immune response to pathogenic bacteria (Hu et al., 2013; Mclamb et al., 2013). The intestine is not only the main organ for the digestion and absorption of nutrients but also the largest organ in the immune system of animals. It has two critical functions: acting as a selective filter for essential nutrients and acting as a barrier against harmful substances (Karl Kunzelmann, 2002; Blikslager et al., 2007). Intestinal barrier dysfunction can cause intestinal microorganisms and endotoxins to break through the intestinal barrier and enter other organs and the circulatory system, resulting in intestinal infections and diseases, such as inflammatory bowel disease, food allergy, diarrhea, and ischemic disease (Torsten et al., 2001; Turner, 2006).

Zinc is an essential trace element that plays a crucial role in several biological processes (Bonaventura et al., 2015). It is an activator or a component of various enzymes in animals and is involved in intracellular signal transduction and cell proliferation, thereby affecting cellular function, acid–base balance, oxidation resistance, immune capacity, and reproduction (Vallee and Falchuk, 1993; Andreini et al., 2006). Moreover, zinc is beneficial for the regeneration of injured intestinal epithelial tissue and is thus necessary for normal intestinal barrier function (Alam et al., 1994). Dietary supplementation with zinc has been shown to reduce intestinal permeability and prevent the loss of intestinal integrity as a result of weaning, heat stress, malnutrition, and inflammatory bowel disease (Rodriguez et al., 1996; Zhang and Guo, 2009; Fernandez et al., 2014). Given these biological functions, zinc may be an attractive feed additive for improving gut health.

Dietary supplementation with inorganic or organic zinc is a common industry practice for meeting the dietary requirements of animals. Inorganic zinc sources, such as zinc sulphate (ZnSO4), have been used as the main nutrient source in feed for a long time (Sandra et al., 2020). In organic zinc sources, zinc binds to organic ligands, typically an organic acid, amino acid, or protein. Organic zinc sources have a relatively higher bioavailability than inorganic ones, allowing lower concentrations to be added to feed (Pearce et al., 2015; Li et al., 2018). Dietary supplementation with organic zinc has been found to improve the growth performance and intestinal health of animals (Levkut et al., 2017; Song et al., 2017; Yan et al., 2017). Zinc lactate, an organic zinc source, has been used as a new feed additive in production practices; however, the effect of zinc lactate on the intestinal health of weaned piglets has not been systematically studied. Moreover, the effects of zinc lactate on the growth performance and intestinal health of piglets need to be compared with those of other zinc sources commonly used in livestock production. Therefore, the objective of the present study was to systematically assess the effects of dietary zinc sources [ZnSO4, glycine zinc (Gly-Zn) and zinc lactate] on the growth performance, intestinal development, digestion and absorption, and intestinal barrier function of weaned piglets in order to further understand the mechanisms underlying the regulatory effects of different zinc sources on intestinal health.

## Materials and Methods

### Animals, Management, and Diets

ZnSO4 was provided by Chengdu Shuxing Feed Co., Ltd. (No 147 Qingpu Road, Shouan, Sichuan, China). Gly-Zn was provided by Sichuan jilongda Biotechnology Group Co., Ltd. (No 111 Jinxing Road, Guanghan, Sichuan, China). Zinc lactate was provided by Sichuan Animtech Feed Co., Ltd. (No.7 Niusha Road, Chengdu, Sichuan, China).

In total, 96 healthy Duroc × Landrace × Yorkshire (DLY) weaned piglets (28days old) with an initial average body weight of 8.81±0.42kg were randomly divided into four groups, with six replicates per treatment and four pigs per replicate, according to their initial body weight and sex. The dietary treatment groups were as follows: (1) control group, basal diet; (2) ZnSO4 group, basal diet +100mg/kg ZnSO4; (3) Gly-Zn group, basal diet +100mg/kg Gly-Zn; and (4) zinc lactate group, basal diet +100mg/kg zinc lactate. The whole trial lasted for 28days. On days 25–28, the digestion test was performed using acid insoluble ash (AIA) as an endogenous indicator.

The basal diets were formulated by using corn and soybean meal as the main ingredients. The experimental diet was prepared according to the nutrient recommendations of NRC (2012) for pigs weighing 7–11kg (Table 1). No antibiotics were used in any diet. Piglets were penned by replicates in sties (1.6×1.5m^2^). The room temperature and relative humidity were controlled during the experimental period. All the piglets had *ad libitum* access to both feed and water. The health status of each pig was checked once a day.

**Table 1:** Composition and nutrient level of basic diet (air dry basis %).

Ingredient	Content	Composition	Nutrient content
Corn	27.79	Calculated Composition	
Extruded corn	28.61	DE (MJ/kg)	3.55
Dehulled soybean meal	10.33	Crude protein	19.59
Extruded soybean	4.50	Calcium	0.81
Fish meal	0.50	Total phosphorus	0.57
Whey powder	8.00	Available phosphorus	0.37
Soybean protein concentrate	12.00	Lys	1.36
Soybean oil	1.90	Met+Cys	0.75
Sucrose	3.50	Thr	0.79
Limestone	0.91	Trp	0.23
Dicalcium phosphate	0.74	Analyzed composition	
Nacl	0.25	Crude protein	19.70
*L*-Lys-HCl (78%)	0.38	Crude ash	4.80
*DL*-Met (99%)	0.17	Dry matter	90.90
Trp (98%)	0.05	Ether extraction	6.20
Thr (98.5%)	0.02	Zinc^3^	0.002
Chloride choline	0.10		
Vitamin premix^1^	0.05		
Mineral premix^2^	0.20		
Total	100.00		

1 *The premix provides following per kg diet:VA 5512IU, VD_3_2250 IU, VE 24mg, VK_3_ 3mg, VB_2_ 6mg, VB_6_ 3mg, VB_12_ 24μg, folic acid 1.2mg, nicotinic acid 14mg, biotin 150μg, D-pantothenic acid 15mg*.

2 *The premix provides following per kg diet: Fe 100mg, Cu 6mg, Mn 4mg, I 0.14mg, Se 0.3mg*.

3 *Analyzed zinc content of experimental diets were as follows: 0.002% (Control group), 0.013% (ZnSO4 group), 0.012% (Gly-Zn group), 0.012% (Zinc lactate group), respectively*.

### Sample Collection

Using the quartering method, about 150g of the experimental diet was taken for each treatment and stored in a refrigerator at −20°C until the analysis of nutrient contents. Fecal samples were collected from days 25 to 28 of the experiment to determine the apparent total tract digestibility (ATTD). After each collection, a few drops of toluene and 10% hydrochloric acid were added to the samples for antisepsis and nitrogen fixation. The fecal samples collected from days 25 to 28 from each replicate were thoroughly mixed and dried in a forced air oven at 60°C for 72h. Following this, the dried samples were smashed and stored at −20°C to measure the nutrient contents.

At the end of day 28 of the experiment, one piglet with an average body weight was selected in each pen, anesthetized with 10mg/kg body weight of Zoletil 50 (Beijing PET Technology Co., Ltd, Beijing, China), and slaughtered by exsanguination. Then, the abdomen was opened and the intestinal segments were rapidly separated. Following this, the intact duodenum, jejunum, and ileum were taken and stored in 4% paraformaldehyde solution for intestinal morphology analysis and goblet cell number determination. The cecal digesta was then collected into sterile EP tubes for determining the bacteria count. Finally, the jejunal mucosa were separated for measuring intestinal development and barrier-related gene expression levels and immediately stored at −80°C.

### Growth Performance

The body weight of each pig was recorded on days 0 and 28; weighing was performed before the pigs were fed. During the experiment, the amount of feed offered daily and the quantity remaining in the feeder the next morning were accurately recorded for each pen. Feed consumption was calculated as the amount of feed offered daily − the quantity remaining in the feeder the next morning − the amount of waste material. The values were used to calculate the average daily gain (ADG) and average daily feed intake (ADFI). The feed-to-gain ratio (F/G) was calculated on the basis of the ADG and ADFI values.

### Diarrhea Rate

Diarrhea scores of all the piglets were recorded each afternoon for 28days according to the following scoring system: 0=normal, firm feces; 1=possible slight diarrhea, soft and formed feces; 2=moderate diarrhea, unformed and slightly fluid feces, and 3=severe diarrhea, very watery feces (Hart and Dobb, 1988). Pigs with a score of 2 or 3 were considered to have diarrhea. The diarrhea rate was calculated using the following formula: diarrhea rate (%)=number of pigs with diarrhea in each pen/(number of pigs × total observation days)×100 (Huang et al., 2004).

### Histological Measurements

The duodenum, jejunum, and ileum, which were fixed in 4% paraformaldehyde solution for 8–24h, were dehydrated, made transparent, and embedded to prepare paraffin sections with a thickness of 5μm. Following this, the sections of the intestinal samples were stained with hematoxylin and eosin. Then, 10 well-oriented slices were selected for each sample and photographed for morphometric variables detection. The distance from the villus tip to the crypt mouth (villus height) and that from the crypt mouth to the base (crypt depth) was measured using an image processing and analysis system (Media Cybernetics, Bethesda, MD, United States). Moreover, the number of jejunal goblet cells was counted using an Olympus optical microscope after histochemical staining with Alcian blue and periodic acid-Schiff (Ab-PAS) stains (Kunert et al., 2002).

### Apparent Total Tract Digestibility

Samples of the feed and feces were assessed to measure the contents of zinc (Chinese National Standard, GB/T 13885-2017), crude ash (method 942.05, AOAC, 1995), CP (method 990.03, AOAC, 1995), EE (method 945.16, AOAC, 1995), and DM (method 930.15, AOAC, 1995). The ATTD was measured using AIA as an endogenous indicator. AIA in diets and feces samples were determined by a method described by Chinese National Standard (GB/T 23742). ATTD was calculated using previously published formulae (Diao et al., 2017).

### Total RNA Extraction, Reverse Transcription Reaction, and Real-Time Quantitative PCR

Total RNA was separated from frozen jejunal mucosa using TRIzol reagent (Takara Bio Inc., Dalian, China), according to the manufacturer’s instructions. Following this, the RNA quality and purity were assessed by electrophoresis on 0.1% agarose gels. Subsequently, eligible RNA samples were reverse transcribed into complementary DNA using the PrimeScript^™^ Reverse Transcription Reagent Kit (Takara Bio Inc., Dalian, China). For the quantification of intestinal development-related genes (IGF-1, insulin-like growth factor 1; EGF, epidermal growth factor), cell apoptosis-related genes (Bcl-2, B-cell lymphoma/leukemia-2; Bax, Bcl-2-associated X protein), intestinal digestion- and absorption-related genes (SGLT-1, sodium/glucose cotransporter 1; GLUT-2, glucose transporter type 2; SLC_7_A_1_, solute carrier family 7; ZNT1, zinc transporter 1; ZNT2, zinc transporter 2; ZIP4, ZRT/IRT-like protein 4), and intestinal barrier-related genes (MUC1, mucin 1; MUC2, mucin 2; occludin; IL-10, interleukin-10; IL-1β, interleukin-1β), real-time PCR was performed using the CFX96 Real-Time PCR Detection System (Bio-Rad Laboratories, Richmond, CA, United States), according to a previously published method (Zhao et al., 2015). A 10μl quantitative fluorescent PCR reaction volume was used in the present study; it consisted of 0.5μl upstream primer, 0.5μl downstream primer, 1μl cDNA, 3μl RNase-free H_2_O, and 5μl SYBR Premix Ex Taq™. The reaction cycle conditions were as follows: 30s at 95°C, 10s at 95°C, and 25s at 60°C for a total of 39cycles. Primer sequences are shown in Table 2; the primers were commercially synthesized by Invitrogen (Shanghai, China). The relative expression level of each gene in the jejunum was computed using β-actin as the reference gene.

**Table 3:** Primes and probes for real-time PCR of bacteria.

Items	Primer/probe name and sequence(5ʹ-3ʹ)	Product length/bp
*Escherichia coli*	DC-F,CATGCCGCGTGTATGAAGAA	96
	DC-R,CGGGTAACGTCAATGAGCAAA
	DC-P,(FMA)AGGTATTAACTTTACTCCCTTCCTC(BHQ-1)	
*Lactobacillus*	RS-F,GAGGCAGCAGTAGGGAATCTTC	126
	RS-R,CAACAGTTACTCTGACACCCGTTCTTC
	RS-P,(FMA)AAGAAGGGTTTCGGCTCGTAAAACTCTGTT(BHQ-1)	
*Bifidobacterium*	SQ-F,CGCGTCCGGTGTGAAAG	121
	SQ-R,CTTCCCGATATCTACACATTCCA
	SQ-P, (FMA) ATTCCACCGTTACACCGGGAA(BHQ-1)	
*Bacillus*	YB-F,GCAACGAGCGCAACCCTTGA	92
	YB-R,TCATCCCCACCTTCCTCCGGT
	YB-P, (FMA)CGGTTTGTCACCGGCAGTCACCT(BHQ-1)	
Total bacteria	Eub338F,ACTCCTACGGGAGGCAGCAG	200
	Eub518R,ATTACCGCGGCTGCTGG

### Microbial Population Determination

Digesta from the cecum were collected, and bacterial DNA was extracted using commercial stool DNA kits (Omega Bio-Tek, Doraville, CA, United States). Fluorescent oligonucleotide probes and primers for total bacteria, *Lactobacillus* spp., *Escherichia coli*., *Bifidobacterium* spp., and *Bacillus* spp. were acquired in accordance with previous reports (Table 3) for the quantitative detection of the aforementioned bacteria (Fierer et al., 2005; Qi et al., 2011), which were commercially synthesized by Invitrogen (Shanghai, China). Quantitative real-time PCR was performed using the CFX96 Real-Time PCR Detection System (Bio-Rad Laboratories, Richmond, CA, United States) with optical-grade 96-well plates. A 25μl reaction mixture was used to determine the total bacterial count, and a 20μl reaction mixture was used to determine the counts of *Lactobacillus* spp., *E. coli*., *Bifidobacterium* spp., and *Bacillus* spp. The PCR conditions and calculation method were in accordance with those reported in a previous study (Qi et al., 2011).

**Table 2:** Primer sequences and annealing temperature of pigs.

Target gene	Forward and reverse primer (5ʹ –3ʹ)	Product length	Annealing temperature	Accession number
EGF	F:ATCTCAGGAATGGGAGTCAACC R: TCACTGGAGGATGGAATACAGC	165	60	NM_214020.1
IGF-1	F:CTGAGGAGGCTGGAGATGTACT R: CCTGAACTCCCTCTACTTGTGTTC	137	60	NM_001097417.1
Bax	F:AAGCGCATTGGAGATGAACT R: TGCCGTCAGCAAACATTTC	121	60	XM_013998624.1
Bcl-2	F:TGCCTTTGTGGAGCTGTATG R: GCCCGTGGACTTCACTTATG	144	60	XM_003121700.4
SGLT-1	F:GCAACAGCAAAGAGGAGCGTAT R: GCCACAAAACAGGTCATAGGTC	137	60	NM_001164021.1
GLUT-2	F:GACACGTTTTGGGTGTTCCG R: GAGGCTAGCAGATGCCGTAG	149	60	NM_001097417.1
SLC_7_A_1_	F:TCTTTGCAGGTCGTTTGGGA R: GGCTGATCACCTGTTGGAGT	137	60	NM_001012613.1
DMT1	F:GCAGGTGGTTGACGTCTGTA R: CACGCCCCCTTTGTAGATGT	100	60	NM_001128440.1
ZNT1	F:TGCTCTGCATGCTGTTACTGA R: TGGAAGGAGTCCGAGAGCAT	97	60	NM_001139470.1
ZNT2	F:GAGATGTGATCGTGGTGCTGATG R: CGCCCAGATATGCAGGTTGTGC	119	60	NM_001139475.1
ZIP4	F:CAGGGTCATCTGGGAAAGGAAGC R: CCGGCACTCAGGCACATCGTG	101	60	XM_001925360.3
Occludin	F:CAGGTGCACCCTCCAGATTG R: GGACTTTCAAGAGGCCTGGAT	110	60	NM_001163647.2
MUC1	F:GTGCCGCTGCCCACAACCTG R: AGCCGGGTACCCCAGACCCA	141	60	XM_001926883.5
MUC2	F:GGTCATGCTGGAGCTGGACAGT R: TGCCTCCTCGGGGTCGTCAC	181	60	XM_013989745.1
IL-10	F:CCTGGAAGACGTAATGCCGA R: CACGGCCTTGCTCTTGTTTT	148	60	NM_214041.1
IL-1β	F:ACGTGCAATGATGACTTTGTCTG R: AGAGCCTTCAGCATGTGTGG	113	60	NM_214055.1
β-actin	F:TCTGGCACCACACCTTCT R: TGATCTGGGTCATCTTCTCAC	114	60	DQ178122

### Statistical Analysis

The experimental data have been tested for normality prior to one-way ANOVA was made using SAS software (version 8.2, SAS Inst. Inc., Cary, NC). When the data were recognized as normally distributed and exhibited homogeneity of variance, data were analyzed by one-way ANOVA and Duncan’s multiple comparison. For data analysis, each pen was considered as an experimental unit. *p* values <0.05 were considered statistically significant. Results are expressed as the means and SEMs.

## Results

### Growth Performance and Diarrhea Rate

The effects of dietary zinc sources on the growth performance and diarrhea rate in weaned piglets are shown in Table 4. Compared with the control group, the F/G decreased in the Gly-Zn and zinc lactate groups from days 0 to 28 (*p*<0.05). However, a lower diarrhea rate was observed only in the zinc lactate group (*p*<0.05). The ADG tended to be higher in the ZnSO4, Gly-Zn, and zinc lactate groups than in the control group (*p*=0.053). No differences were observed in the ADFI among the four groups during the experimental period (*p*>0.05).

**Table 4:** Effect of dietary zinc sources on the growth performance and diarrhea rate in weaned piglets.

Items	Control	ZnSO4	Gly-Zn	Zinc lactate	SEM	*P* values
Initial BW, kg	8.68	8.92	8.86	8.82	0.212	0.873
28-day BW, kg	17.25	18.31	17.87	17.89	0.441	0.444
ADFI (g)	517.41	534.41	496.54	508.78	16.040	0.442
ADG (g)	306.16	335.68	321.99	334.02	7.034	0.053
F/G	1.69^a^	1.60^ab^	1.54^b^	1.52^b^	0.031	0.021
Diarrhea rate	20.24^a^	17.86^ab^	13.53^ab^	10.04^b^	2.217	0.041

*Control, basal diet. ZnSO4, basal diet+100mg/kg zinc sulphate. Gly-Zn, basal diet+100mg/kg glycine zinc. Zinc lactate, basal diet+100mg/kg zinc lactate. BW, body weight. ADFI, average daily feed intake. ADG, average daily gain. F/G, feed-to-gain ratio. SEM, standard error of the mean. ^a,b^, Within a row, means without a common superscript letter differ (P<0.05)*.

### Apparent Total Tract Digestibility

The effects of dietary zinc sources on the ATTD in weaned piglets are shown in Table 5. The ATTD of DM, CP, EE, and crude ash was significantly higher in the ZnSO4, Gly-Zn, and zinc lactate groups than in the control group (*p*<0.05). The ATTD of zinc significantly differed among the three groups receiving dietary zinc supplementation; it was the highest in the zinc lactate group, followed by the Gly-Zn and ZnSO4 groups (*p*<0.05).

**Table 5:** Effect of dietary zinc sources on the apparent total tract digestibility in weaned piglets.

Items	Control	ZnSO4	Gly-Zn	Zinc lactate	SEM	*P* values
EE	71.32^b^	76.44^a^	77.72^a^	78.84^a^	0.954	0.002
DM	84.56^b^	87.59^a^	87.60^a^	87.71^a^	0.344	0.001
Crude ash	60.55^b^	67.64^a^	69.45^a^	70.12^a^	0.588	<0.0001
CP	77.35^b^	80.97^a^	80.63^a^	81.73^a^	0.544	0.001
Zinc	2.08^c^	10.27^c^	27.91^b^	42.95^a^	2.212	<0.0001

*Control, basal diet. ZnSO4, basal diet+100mg/kg zinc sulphate. Gly-Zn, basal diet+100mg/kg glycine zinc. Zinc lactate, basal diet+100mg/kg zinc lactate. EE, ether extract; DM, dry matter; CP, crude protein. SEM, standard error of the mean. ^a,b^, Within a row, means without a common superscript letter differ (P<0.05)*.

### Relative mRNA Expression Levels of Jejunal Transporters

**Figure 1:**
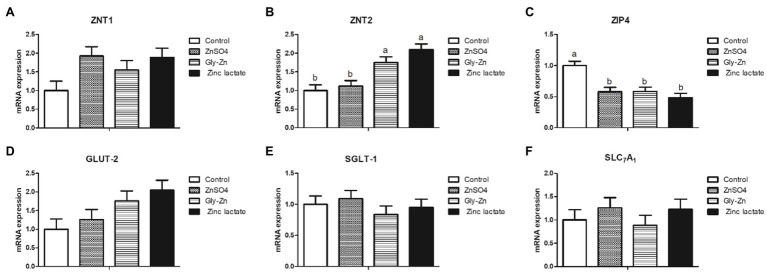
Effect of dietary zinc sources on the relative mRNA expression levels of jejunal digestion- and absorption-related genes in weaned piglets. Control, basal diet. ZnSO4, basal diet +100mg/kg zinc sulphate. Gly-Zn, basal diet +100mg/kg glycine zinc. Zinc lactate, basal diet +100mg/kg zinc lactate. The relative mRNA expression levels of jejunal zinc transporter 1 (ZNT1; **A)**, zinc transporter 2 (ZNT2; **B)**, ZRT/IRT-like protein 4 (ZIP4; **C)**, glucose transporter type 2 (GLUT-2; **D)**, sodium/glucose cotransporter 1 (SGLT-1; **E)**, and solute carrier family 7 (SLC_7_A_1_; **F)** were determined by real-time quantitative PCR. ^a,b^ Within a row, means without a common superscript letter differ (*p*<0.05).

As shown in Figure 1, the mRNA expression level of jejunal ZIP4 was significantly lower in the ZnSO4, Gly-Zn, and zinc lactate groups than in the control group (*p*<0.05). The mRNA expression of jejunal ZNT2 was higher in the Gly-Zn and zinc lactate groups than in the control group (*p*<0.05). However, the mRNA expression level of jejunal ZNT2 did not differ significantly between the ZnSO4 and control groups (*p*>0.05). The mRNA expression level of jejunal ZNT1 tended to be higher in the ZnSO4, Gly-Zn, and zinc lactate groups than in the control group (*p*=0.067). However, the mRNA expression level of jejunal GLUT-2 tended to be higher only in the Gly-Zn and zinc lactate groups than in the control group (*p*=0.064). Moreover, the mRNA expression levels of jejunal SGLT-1 and SLC_7_A_1_ did not differ among the four groups (*p*>0.05).

### Intestinal Morphology

**Figure 2:**
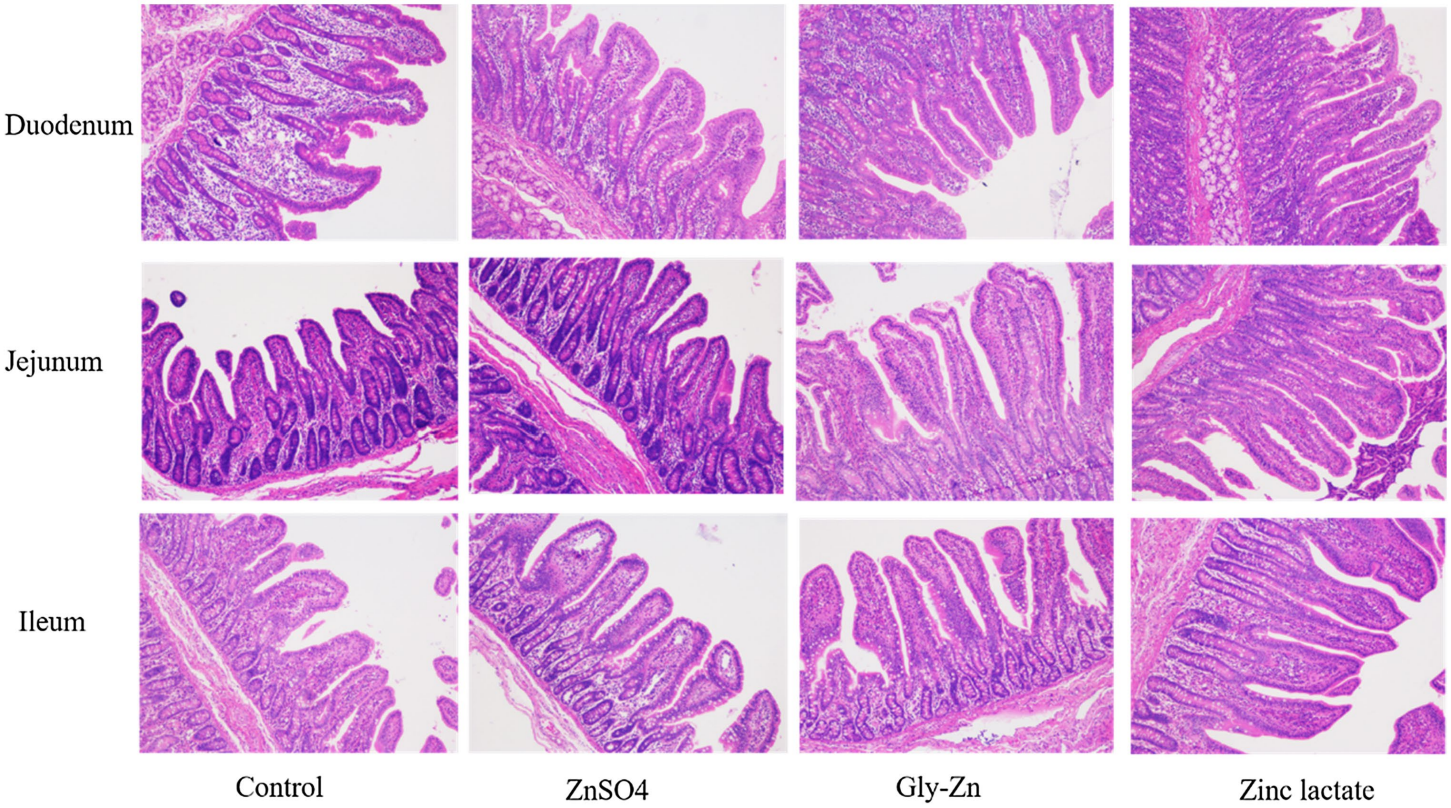
Effect of dietary zinc sources on intestinal morphology (images) in weaned piglets. Control, basal diet. ZnSO4, basal diet +100mg/kg zinc sulphate. Gly-Zn, basal diet +100mg/kg glycine zinc. Zinc lactate, basal diet +100mg/kg zinc lactate.

The villus height, crypt depth, and goblet cell number in small intestinal tissues are expressed in Table 6 and Figure 2. The jejunal villus height and villus height:crypt depth ratio were higher in the Gly-Zn and zinc lactate groups than in the control group (*p*<0.05). The numbers of goblet cells in the ileum (*p*=0.064) and the villus height of the duodenum (*p*=0.094) and ileum (*p*=0.060) tended to be higher in the zinc lactate group than in the control group. In addition, the jejunal crypt depth tended to be lower in the ZnSO4 and zinc lactate groups than in the control group (*p*=0.079).

**Table 6:** Effect of dietary zinc sources on the intestinal morphology and number of goblet cells in weaned piglets.

Items	Control	ZnSO4	Gly-Zn	Zinc lactate	SEM	*P* values
**Duodenum**						
Villus height, μm	352.75	381.74	412.31	442.35	24.132	0.094
Crypt depth, μm	145.74	139.89	142.07	144.08	6.417	0.925
Villus height: crypt depth	2.59	2.74	2.91	2.95	0.206	0.602
**Jejunum**						
Villus height, μm	332.86^b^	354.33^ab^	414.85^a^	416.92^a^	16.507	0.004
Crypt depth, μm	159.69	125.63	140.91	126.22	9.649	0.079
Villus height: crypt depth	2.46^b^	2.85^ab^	2.95^a^	3.21^a^	0.117	0.004
**Ileum**						
Villus height, μm	309.84	332.10	321.14	382.55	18.325	0.060
Crypt depth, μm	146.44	148.79	147.40	139.51	7.486	0.822
Villus height: crypt depth	2.66	2.78	2.69	3.07	0.132	0.152
Goblet cells	316.56	339.56	330.67	387.72	17.856	0.064

*Control, basal diet. ZnSO4, basal diet+100mg/kg zinc sulphate. Gly-Zn, basal diet+100mg/kg glycine zinc. Zinc lactate, basal diet+100mg/kg zinc lactate. SEM, standard error of the mean. ^a,b^, Within a row, means without a common superscript letter differ (P<0.05)*.

### Relative mRNA Expression Levels of Intestinal Development-Related Genes

**Figure 3:**
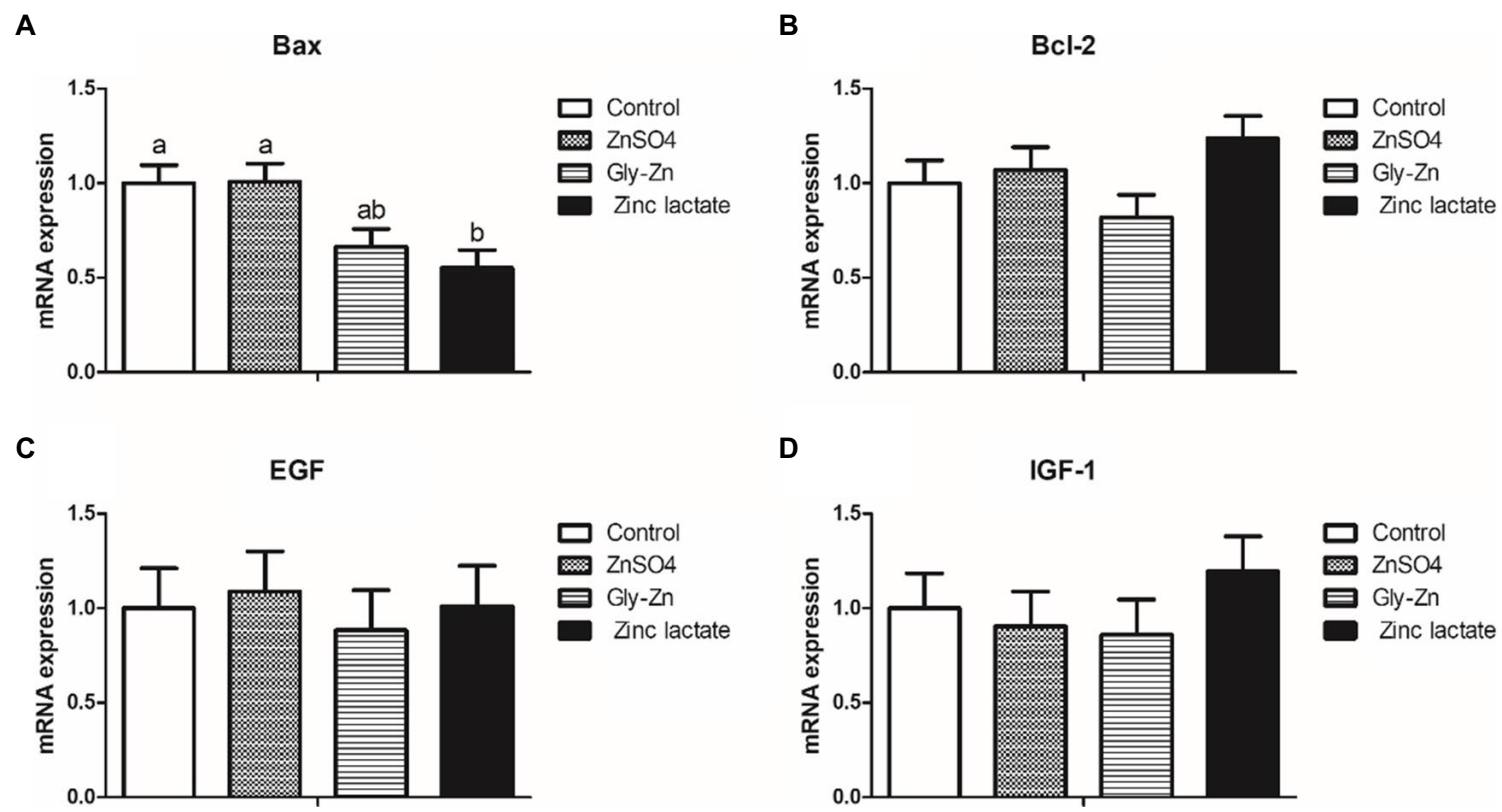
Effect of dietary zinc sources on the relative messenger RNA (mRNA) expression levels of intestinal development-related genes in weaned piglets. Control, basal diet. ZnSO4, basal diet +100mg/kg zinc sulphate. Gly-Zn, basal diet +100mg/kg glycine zinc. Zinc lactate, basal diet +100mg/kg zinc lactate. The relative mRNA expression levels of jejunal Bcl-2-associated X protein (Bax; **A)**, B-cell lymphoma/leukaemia-2 (Bcl-2; **B)**, epidermal growth factor (IGF-1; **C)**, and insulin-like growth factor-1 (IGF-1; **D)** were determined by real-time quantitative PCR. ^a,b^ Within a row, means without a common superscript letter differ (*p*<0.05).

The mRNA expression levels of intestinal development-related genes in the piglets are summarized in Figure 3. The Gly-Zn and zinc lactate groups had a lower mRNA expression level of jejunal Bax than the control group (*p*<0.05). However, the mRNA expression level of jejunal Bax did not differ significantly between the ZnSO4 and control groups (*p*>0.05). Moreover, the mRNA expression levels of jejunal Bcl-2, EGF, and IGF-1 did not differ among the four groups (*p*>0.05).

### Intestinal Barrier Function

**Figure 4:**
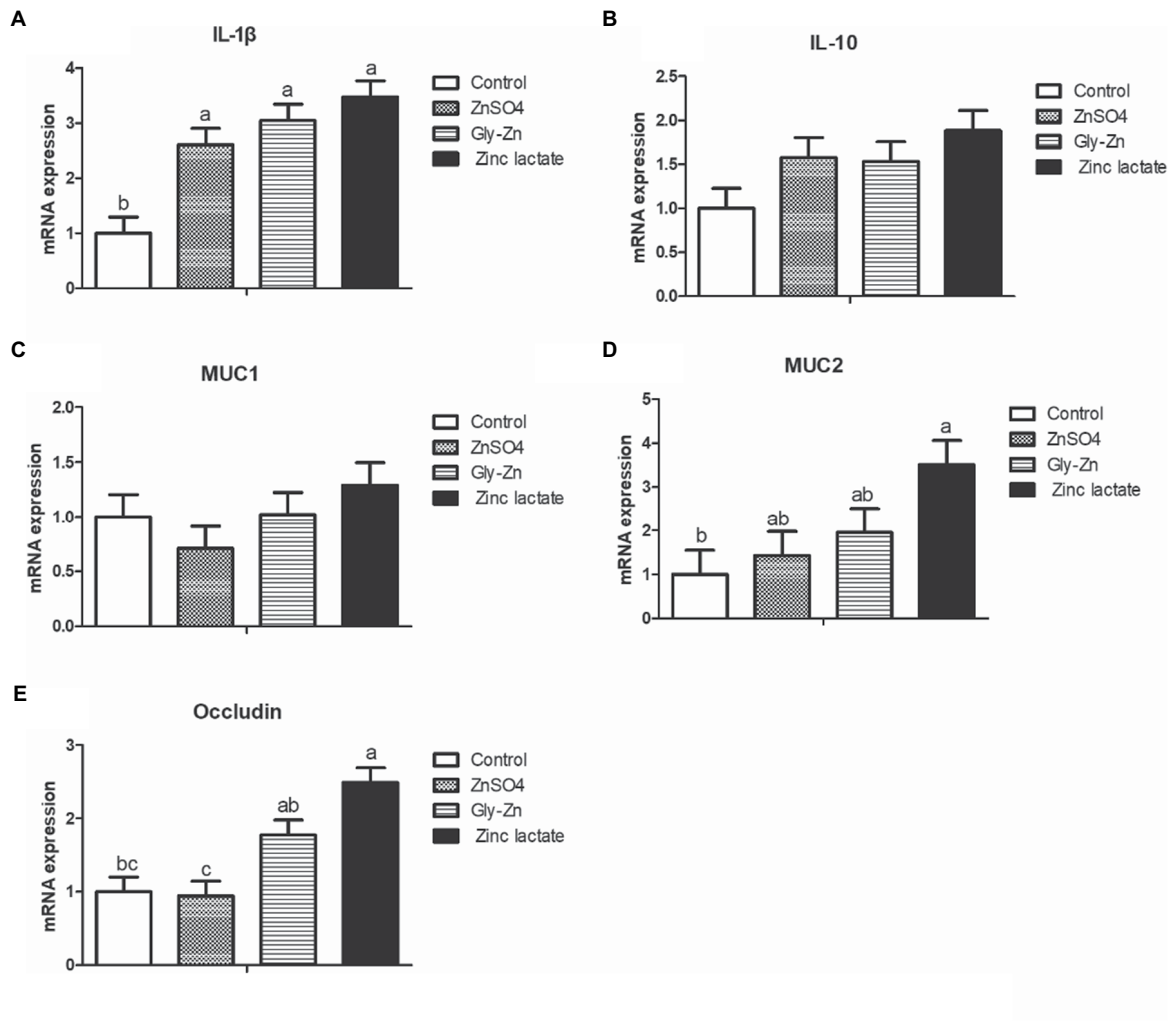
Effect of dietary zinc sources on the relative mRNA expression levels of jejunal barrier-related genes in weaned piglets. Control, basal diet. ZnSO4, basal diet +100mg/kg zinc sulphate. Gly-Zn, basal diet +100mg/kg glycine zinc. Zinc lactate, basal diet +100mg/kg zinc lactate. The relative mRNA expression levels of jejunal interleukin-1β (IL-1β; **A)**, interleukin-10 (IL-10; **B)**, mucin 1 (MUC1; **C)**, mucin 2 (MUC2; **D)**, and Occludin; **E)** were determined by real-time quantitative PCR. ^a,b^ Within a row, means without a common superscript letter differ (*p*<0.05).

As shown in Figure 4, the zinc lactate group had a higher mRNA expression level of the jejunal occludin gene than the control group (*p*<0.05). Moreover, the Gly-Zn and zinc lactate groups had a higher mRNA expression level of the jejunal occludin than the ZnSO4 group (*p*<0.05). The mRNA expression level of jejunal IL-1β was higher in the ZnSO4, Gly-Zn, and zinc lactate groups than in the control group (*p*<0.05). In addition, the mRNA expression level of jejunal IL-10 tended to be higher in the ZnSO4, Gly-Zn, and zinc lactate groups than in the control group (*p*=0.087). The mRNA expression level of jejunal MUC2 was higher in the zinc lactate group than in the control group (*p*<0.05). However, the mRNA expression level of jejunal MUC2 did not differ significantly among the ZnSO4, Gly-Zn, and control groups (*p*>0.05).

As shown in Table 7, the total bacterial count in the cecal digesta was higher in the ZnSO4, Gly-Zn, and zinc lactate groups than in the control group (*p*<0.05). The zinc lactate group had a higher count of *Lactobacillus* spp. in the cecal digesta than the control group (*p*<0.05). However, the count of *E. coli* in the cecal digesta tended to be lower in the Gly-Zn and zinc lactate groups than in the control group (*p*=0.075).

**Table 7:** Effect of dietary zinc source on the numbers of *Escherichia coli*, *Lactobacilli* spp., *Bifidobacterium* spp., and *Bacillus* spp. in the cecal digesta of weaned piglets (log [copies/g]).

Items	Control	ZnSO4	Gly-Zn	Zinc lactate	SEM	*P* values
Total bacteria	9.90^b^	11.05^a^	11.04^a^	11.53^a^	0.276	0.006
*Bacillus* spp.	8.17	8.60	8.63	8.50	0.140	0.126
*Lactobacillus* spp.	6.79^b^	6.93^b^	7.50^ab^	8.03^a^	0.177	0.001
*Escherichia coli*	9.72	9.77	9.14	9.20	0.198	0.075
*Bifidobacterium* spp.	5.28	5.72	5.65	5.81	0.290	0.601

*Control, basal diet. ZnSO4, basal diet+100mg/kg zinc sulphate. Gly-Zn, basal diet+100mg/kg glycine zinc. Zinc lactate, basal diet+100mg/kg zinc lactate. SEM, standard error of the mean. ^a,b^ Within a row, means without a common superscript letter differ (p<0.05)*.

## Discussion

Zinc is a component of various enzymes in animals; it has important physiological and nutritional functions for animal growth, reproduction, and immunity. It also exhibits cell growth-promoting and antioxidant effects (Bonaventura et al., 2015). Zinc depletion tests have confirmed that zinc deficiency can result in lower ADG, decreased growth hormone synthesis, and reduced production of IGF-1 induced by growth hormone, thereby impairing the growth of piglets (Swinkels et al., 1996). In the present study, the ADG in the zinc supplementation groups (ZnSO4, Gly-Zn, and zinc lactate groups) was higher than that in the control group, indicating a beneficial effect of zinc on growth performance. On comparing different zinc sources, organic zinc sources were found to have a relatively higher bioavailability than inorganic ones (Pearce et al., 2015; Li et al., 2018). In a previous study, weaned piglets fed 20–120mg Zn/kg in an organic form (zinc amino acid [ZnAA]) were found to have a lower F/G than those in the unsupplemented group, while no differences were noted between the inorganic zinc-supplemented and unsupplemented groups (Zhang et al., 2017). Under nursery conditions, dietary supplementation with 500mg/kg polysaccharide zinc complex had the same effect as that supplemented with 3,000mg/kg pharmacological zinc oxide on enhancing the growth performance of piglets (Case and Carlson, 2002). In another study, broilers fed diets supplemented with 60mg/kg ZnAA complexes had a lower F/G in the starter phase than those fed diets supplemented with ZnSO4 (Chand et al., 2020; Grande et al., 2020). Similarly, the organic form of zinc (zinc lactate) was found to be superior in improving the growth of young grass carp (Song et al., 2017). In the present study, compared with the control group, the F/G decreased in the organic zinc-supplemented groups (Gly-Zn and zinc lactate groups) from days 0 to 28; these findings are generally consistent with those of the aforementioned studies. Moreover, zinc deficiency could alter paracellular ionic conductance, cause perturbed barrier integrity and reduce Cl^−^ secretion, resulting in increased susceptibility to infection (Sarkar et al., 2018). Thus, adequate levels are required to maintain the gut barrier, avoid risk intestinal infections, and prevent diarrhea. However, a lower diarrhea rate was observed only in the zinc lactate group in the present study, suggesting that zinc lactate has a more beneficial effect on intestinal health than Gly-Zn.

In the present study, the ATTD of DM, CP, EE, and crude ash was found to be increased in pigs fed 100mg/kg zinc, regardless of the zinc source. However, the ATTD of zinc significantly differed among the three groups receiving dietary zinc supplementation; it was the highest in the zinc lactate group, followed by the Gly-Zn and ZnSO4 groups. In general, the improvement in nutrient digestibility is accompanied by the elevation in growth performance; this was mutually confirmed by improved growth performance in the present study. Consistent with our result, dietary supplementation with 60mg/kg ZnAA complexes was found to result in better digestibility of zinc than supplementation with ZnSO4 in young broilers in a previous study (Zhang et al., 2017). Supplementation with different zinc sources (zinc lactate, Gly-Zn, and ZnSO4) did not affect the digestibility of other nutrients (DM, CP, EE, and crude ash) in the present study; these findings are in accordance with those of previous studies comparing the supplementation of Gly-Zn and ZnSO4 (Ma et al., 2011; Kwiecień et al., 2017). The ZnT family, which is responsible for decreasing the concentration of zinc ions in the cytoplasm, and the ZIP family, which is responsible for increasing the concentration of zinc ions in the cytoplasm, play important roles in the absorption and transport of zinc ions (Nies, 2007). In the present study, dietary supplementation with 100mg/kg ZnSO4, Gly-Zn, or zinc lactate decreased the mRNA expression level of jejunal ZIP4 in weaned piglets. The mRNA expression level of jejunal ZNT2 was higher in the Gly-Zn and zinc lactate groups than in the control group; these findings are consistent with those of a previous *in vitro* study (Huang et al., 2016). A study conducted using a pig model also revealed that the addition of zinc lactate to the medium could upregulate the mRNA expression level of ZNT2 and downregulate the mRNA expression level of ZIP4 (Wang et al., 2014). It has been reported that zinc has acquired an insulin-like activity (Tang and Shay, 2001). A higher mRNA expression level of jejunal GLUT-2 was observed in the Gly-Zn and zinc lactate groups in the present study, indicating that organic zinc may stimulate higher glucose transporter expression and lower blood glucose levels.

Weaning stress disturbs the intestinal health balance, which is characterized by villous atrophy and crypt hyperplasia, in addition to a reduction in epithelial brush border activity and nutrient digestibility (Montagne et al., 2003; Wang et al., 2006). Consequently, maintaining intestinal morphological properties for digesting various nutrients after weaning is important. In the present study, the jejunal villus height and villus height:crypt depth ratio were higher in the Gly-Zn and zinc lactate groups than in the control group. Moreover, the villus heights of the duodenum and ileum were higher in the zinc lactate group than in the control group. In accordance with our findings, dietary supplementation with 100mg/kg zinc lactate was found to significantly increase the villus height and decrease the crypt depth of the duodenum, jejunum, and ileum in weaned piglets in a previous study, thereby improving the morphology and function of the intestinal epithelium (Wang et al., 2014). Similarly, dietary supplementation with 90mg/kg Gly-Zn increased the villus height of the duodenum and jejunum and decreased the crypt depth of the jejunum and ileum in 42-day-old chickens in another study (Ma et al., 2011). Intestinal morphology is directly proportional to digestibility and, consequently, to feed conversion efficiency (Collett, 2012). Therefore, the improved intestinal morphology may partly explain the lowered F/G and increased digestibility of zinc on dietary supplementation with Gly-Zn or zinc lactate. On the other hand, the improvement in intestinal epithelial morphology caused by supplementation with organic zinc may be related to the promotion of intestinal epithelial cell development. *In vitro*, zinc lactate could significantly promote the proliferation of the porcine jejunal epithelial cells IPEC-J2 after 48h of inoculation, and the proliferation amplitude was elevated with an increase in zinc lactate concentrations (Han et al., 2012). Meanwhile, zinc lactate could significantly reduce the cell apoptosis rate and the expression level of apoptotic protein in IPEC-J2 cells induced by hydrogen peroxide (Tang et al., 2020). In our present study, a lower mRNA expression level of jejunal *Bax* (a pro-apoptotic gene) was observed in the zinc lactate group, indicating that zinc lactate may promote intestinal morphology by promoting cell proliferation and inhibiting cell apoptosis.

Intestinal barrier integrity is primarily maintained by the tight junctions. Zinc deficiency causes the release of zinc bound to proteins and increases the content of free zinc in the cytoplasm, resulting in the inhibition of cell growth, disruption of tight junction proteins, and the subsequent impairment of intestinal barrier function (Zhong et al., 2010). In an *in vitro* study, compared with ZnSO4, 300μmol zinc butyrate could increase transmembrane resistance and maintain the integrity of tight junctions in IPEC-J2 cells, indicating that zinc butyrate could alleviate the increased permeability of IPEC-J2 cells induced by heat stress (Mani et al., 2019). Sanz Fernandez et al. (2014) revealed that the transepithelial resistance of the ileum was 56% higher in the ZnAA complex group than in the ZnSO4 group (Fernandez et al., 2014). In grass carp, compared with the 56.9mg/kg ZnSO4 group, dietary supplementation with 49.84mg/kg zinc lactate upregulated the mRNA expression levels of occludin, ZO-1, claudin-B, claudin-C, claudin-F, claudin-3, claudin-7A, claudin-7B, claudin-11, claudin-12, and claudin-15A genes in the intestine (Song et al., 2017). Consistent with previous findings, we found that the mRNA expression level of the jejunal occludin was higher in the zinc lactate group than in the control and ZnSO4 groups. Myosin light-chain kinase (MLCK) is the most important calmodulin kinase affecting the barrier function of the intestinal mucosal epithelium (Nalle et al., 2011). Tumor necrosis factor-α (TNF-α) induces the expression of MLCK and phosphorylated myosin light chains, resulting in a loss of intercellular tight junctions and further increasing intestinal epithelial permeability through paracellular pathways (Mckenzie and Ridley, 2007). Previous studies have demonstrated that organic zinc can downregulate the expression of TNF-α in the intestine (Li, 2015; Song et al., 2017). Therefore, it is speculated that organic zinc can maintain the normal tight junctions of intestinal epithelial cells by inhibiting the TNF-α-induced upregulation of MLCK expression.

Among the chemical barrier components of the intestinal mucosa, MUC2, which is mainly secreted by cup cells, is the main component of intestinal mucus and plays an important role in lubricating the intestinal tract, in providing adhesion sites for intestinal antibacterial proteins and symbiotic flora and in resisting the invasion of intestinal pathogens and harmful substances (Hasnain et al., 2010). Compared with ZnSO4, protein-chelated zinc was found to significantly increase the number of intraepithelial goblet cells in the duodenum and jejunum of growing-finishing pigs (Zhou, 2009). In a previous study, dietary supplementation with 30mg/kg Gly-Zn or ZnSO4 upregulated the mRNA expression level of jejunal *MUC2* in broilers. The mRNA expression level of *MUC2* in the Gly-Zn group tended to be higher than that in the ZnSO4 group (Levkut et al., 2017); these findings are in accordance with those of the present study. In the present study, supplementation with zinc lactate increased the number of goblet cells in the ileum and upregulated the transcription level of *MUC2* in the intestine, indicating that zinc lactate could also improve the intestinal chemical barrier function in weaned piglets.

The intestinal tract is the largest organ in the immune system of animals, and maintaining normal intestinal barrier function is extremely important for good intestinal health (Chassaing et al., 2014). Long-term zinc deficiency leads to the infiltration of inflammatory cells, particularly the uncontrolled migration of epithelial multinucleated lymphocytes, in the intestinal mucosa, thereby inducing mucosal injury and damaging the intestinal immune barrier (Finamore et al., 2008). Moreover, zinc deficiency can reduce the proliferation of T lymphocytes and B lymphocytes in mice, weaken their ability to deal with the invasion of exogenous pathogenic microorganisms and damage their immune system (Srinivas et al., 1989). Cytokines have momentous effects on immune responses and are involved in regulating intestinal barrier integrity (Al-Sadi, 2009). Zinc promotes the adhesion of monocytes to endothelial cells; this is important for the production of inflammatory cytokines, such as IL-1β, IL-6, and TNF-α (Chavakis et al., 1999). Consistently, higher mRNA expression levels of jejunal *IL-1β* and *IL-10* were observed following zinc supplementation in the present study, indicating that dietary supplementation with 100mg/kg zinc can improve intestinal immune function in weaned piglets. Very limited studies have assessed the effects of different zinc sources on the intestinal immune barrier. In grass carp, compared with the 56.9mg/kg ZnSO4 group, dietary supplementation with 49.84mg/kg zinc lactate was found to upregulate the mRNA expression level of *IL-10* in the intestine (Song et al., 2017). However, there was no difference in the effect of different zinc sources on inflammatory cytokines; this could be related to zinc levels, animal species, period, and health status, among others.

The digestive tract of animals has evolved into a key site for the coexistence of nutrients and microorganisms. The intestinal biological barrier is formed by a large number of normal microflora, and the intestinal microecological balance is crucial for the normal function of the intestinal biological barrier (Shanahan, 2002). Zinc is an essential mineral element involved in the colonization and proliferation of microorganisms in the host. Chronic dietary zinc depletion induces significant taxonomic alterations in the intestinal microflora and decreases the overall species richness and diversity, thereby establishing a microbial profile resembling that of various pathological states (Spenser et al., 2015). In the present study, dietary supplementation with 100mg/kg zinc increased the total bacterial count in the cecal digesta of weaned piglets. As we did not assess the microbial diversity in the cecal digesta, the effects of different zinc sources on the intestinal microbial diversity need to be studied further. Another intriguing finding of the present study was that the count of *Lactobacillus* spp. in the cecal digesta was higher in the zinc lactate group than in the control group. Moreover, the count of *E. coli* in the cecal digesta was lower in the Gly-Zn and zinc lactate groups than in the control group. The alteration in and balance between beneficial bacteria (such as *Lactobacillus* spp.) and harmful bacteria (such as pathogenic *E. coli*) in the gut are associated with the gut health of the host (Diao et al., 2015). *E. coli* has been reported to destabilize and dissociate tight junction proteins (Muza-Moons et al., 2004). In the present study, the increased mRNA expression level of the occludin gene observed in the zinc lactate group was in accordance with the decreased *E. coli* count. Collectively, these findings suggest that zinc lactate could maintain the balance of the gut microbiota and improve the intestinal barrier.

## Conclusion

In conclusion, dietary supplementation with 100mg/kg ZnSO4, Gly-Zn, or zinc lactate could improve the growth performance of weaned pigs, at least partly, by improving the digestion of nutrients, intestinal morphology, and barrier function. Dietary supplementation with organic zinc, particularly zinc lactate, was found to have the best effect. However, the mRNA expression levels of zinc transporters were not consistent between the groups receiving dietary supplementation with inorganic and organic zinc, indicating possible differences in the absorption and transport channels.

## Data Availability Statement

The original contributions presented in the study are included in the article/supplementary material, further inquiries can be directed to the corresponding author.

## Ethics Statement

The animal study was reviewed and approved by Animal Care Advisory Committee of Sichuan Academy of Animal Science.

## Author Contributions

HD, JY, and SL conceived the study, designed, and performed the experiments, including chemical analysis, analyzed the experimental data, and wrote the manuscript. WT was responsible for conceptualization. SK and JZ verified the validity of the experiments and checked the results. XW, MZ, CH, and PH participated in the experimental design and gave important intellectual advice for approval. All the authors read and approved the final version of the manuscript.

## Funding

The present study was financially supported by the Sichuan Science and Technology Programmes (2020YFH0170, 2021ZHYZ0005, and 2021NZZJ0019).

## Conflict of Interest

Author SL was employed by company Sichuan Animtech Biology Development Co., Ltd. Author SK was employed by company Sichuan Animtech Feed Co., Ltd. The remaining authors declare that the research was conducted in the absence of any commercial or financial relationships that could be construed as a potential conflict of interest.

## Publisher’s Note

All claims expressed in this article are solely those of the authors and do not necessarily represent those of their affiliated organizations, or those of the publisher, the editors and the reviewers. Any product that may be evaluated in this article, or claim that may be made by its manufacturer, is not guaranteed or endorsed by the publisher.
